# Persistence with oral bisphosphonates and denosumab among older adults in primary care in Ireland

**DOI:** 10.1007/s11657-021-00932-7

**Published:** 2021-04-17

**Authors:** Mary E. Walsh, Tom Fahey, Frank Moriarty

**Affiliations:** 1 HRB Centre for Primary Care Research, Department of General Practice, Royal College of Surgeons in Ireland, Dublin, Ireland; 2 School of Pharmacy and Biomolecular Sciences, Royal College of Surgeons in Ireland, 111 St Stephen’s Green, Dublin 2, Ireland

**Keywords:** Osteoporosis, Bone density conservation agents, Primary healthcare, Medication persistence

## Abstract

***Summary*:**

Gaps in pharmacological treatment for osteoporosis can reduce effectiveness. Among older adults, we found about half of new users of oral bisphosphonate and denosumab persisted with their treatment at 2 years, with few switching to alternative therapy. Persistence is suboptimal and warrants evaluation of interventions to improve this.

**Purpose:**

Gaps in pharmacological treatment for osteoporosis can reduce effectiveness. This study aimed to estimate persistence rates for oral bisphosphonates and denosumab in older primary care patients and identify factors associated with discontinuation.

**Methods:**

Older patients newly prescribed oral bisphosphonates or denosumab during 2012–2017 were identified from 44 general practices (GP) in Ireland. Persistence without a coverage gap of >90 days was calculated for both medications from therapy initiation. Factors associated with time to discontinuation were explored using Cox regression analysis. Exposures included age group, osteoporosis diagnosis, fracture history, calcium/vitamin D prescription, number of other medications, health cover, dosing frequency (bisphosphonates) and previous bone-health medication (denosumab).

**Results:**

Of 41,901 patients, *n*=1569 were newly initiated on oral bisphosphonates and *n*=1615 on denosumab. Two-year persistence was 49.4% for oral bisphosphonates and 53.8% for denosumab and <10% were switched to other medication. Having state-funded health cover was associated with a lower hazard of discontinuation for both oral bisphosphonates (HR=0.49, 95% CI=0.36–0.66, *p*<0.01) and denosumab (HR=0.71, 95% CI=0.57–0.89, *p*<0.01). Older age group, number of medications and calcium/vitamin D prescription were also associated with better bisphosphonate persistence, while having osteoporosis diagnosed was associated with better denosumab persistence.

**Conclusion:**

Persistence for osteoporosis medications is suboptimal. Of concern, few patients are switched to other bone-health treatments when denosumab is stopped which could increase fracture risk. Free access to GP services and medications may have resulted in better medication persistence in this cohort. Future research should explore prescribing choices in primary care osteoporosis management and evaluate cost-effectiveness of interventions for improving persistence.

**Supplementary Information:**

The online version contains supplementary material available at 10.1007/s11657-021-00932-7.

## Introduction

Osteoporosis and associated fragility fractures can result in significant disability, morbidity and mortality with 20% of individuals who experience a hip fracture dying in the first year [1, 2]. It is estimated that one in five women and one in twenty men over the age of 60 have osteoporosis and 3% of older adults are expected to experience fragility fractures annually [3, 4]. Adults at high fracture risk, including those with osteoporosis, those with previous fractures or those who take medication that reduces bone quality, should be offered pharmacological treatment where no contraindication exists [5–8]

Oral bisphosphonates have been shown to prevent fractures in men and women, and they are the most cost-effective initial therapy for osteoporosis [6, 8, 9]. Denosumab, a newer antiresorptive treatment, involves six monthly administration by subcutaneous injection, usually administered by a healthcare professional. It is recommended in patients with high fracture risk where they are unable to take oral bisphosphonates due to difficulties with administration or intolerance caused by upper gastrointestinal symptoms [6–8]. Denosumab has been shown to prevent fractures in women [7]. While research in men remains limited, it improves bone mineral density (BMD) and has shown an effect on fracture incidence in particular cohorts [7, 9, 10]. To be cost-effective and result in optimal fracture reduction, it is important that oral bisphosphonates and denosumab are prescribed and taken/administered correctly, at the appropriate time intervals, without unwarranted gaps in treatment or early cessation [11, 12]. Adherence (the extent to which a patient acts in accordance with the prescribed interval and dose regimen) and persistence (the accumulation of time from initiation to discontinuation of therapy) have both been found to be suboptimal in oral bisphosphonate and denosumab use [13, 14].

Clinical guidelines recommend that bisphosphonates are continued without a break for a period of at least 3 years and for up to 10 years in those deemed to be at high risk of fracture [6, 15–17]. However, a large recently published systematic review found that 2-year persistence for oral bisphosphonates was less than 30% in most studies and that only 35% to 48% of patients are adherent at 2 years [13]. Persistence in denosumab treatment is particularly important, as the suppression of bone resorption rapidly reverses where treatment is delayed by as little as 3 months [18, 19]. There is some evidence that this could result in rebound vertebral fractures [20]. A recent systematic review including 16 studies of denosumab showed that average 2-year persistence was only 55% [14]. Treatment with oral bisphosphonates after stopping denosumab is protective against negative effects in most patients after 1 year of treatment; however, stronger replacement treatments may be required for patients taking denosumab for longer periods [21, 22].

Internationally, general practitioners (GPs) have reported uncertainty about prescription breaks in bisphosphonate treatment and cessation of denosumab [23]. Recent estimates of persistence for these medications are not available in primary care in Ireland, and so the extent of the problem in the Irish setting is unknown. Furthermore, identification of factors associated with early discontinuation of these medications in a large representative primary care database could reveal circumstances in which education or input from specialists would be warranted.

### Study objectives

The aim of this study is to estimate persistence rates for oral bisphosphonates and denosumab in a cohort of older primary care patients in Ireland who are newly prescribed these medications and to identify factors associated with time to discontinuation.

## Methods

### Study design

The REporting of studies Conducted using Observational Routinely-collected health Data (RECORD) statement was used in the conduct and reporting of this retrospective cohort study.[24]

### Setting and data source

Data were collected as part of a larger study from 44 general practices in the Republic of Ireland in the areas of Dublin (*n*=30), Galway (*n*=11) and Cork (*n*=3) using the patient management software Socrates (www.socrates.ie) between January 2011 and 2017 [25, 26]. Data, anonymised at the time of extraction, included demographic, clinical, prescribing and hospitalisation records of patients who were 65 years and older at the date of data extraction (2017). Ethical approval was obtained from the Irish College of General Practitioners.

### Participants

**Fig. 1:**
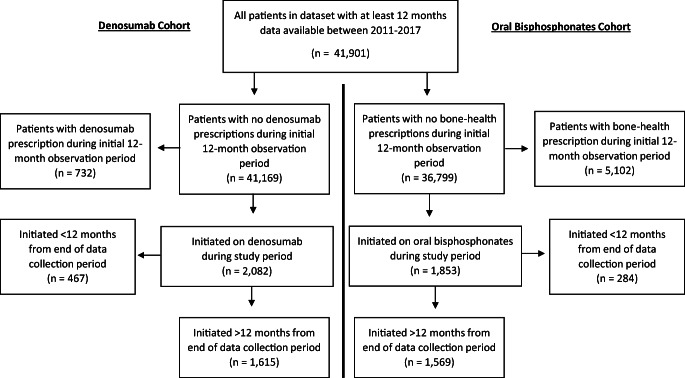
Flow diagram of patient selection

Patients were eligible for inclusion in analysis if they were newly prescribed oral bisphosphonates or denosumab during the study period (see Fig. 1). Cohorts of patients initiated on oral bisphosphonates and denosumab were defined and analysed separately, resulting in potential overlap between these groups.

Prescriptions for bone-health medications were identified from two sources: GP prescription records (WHO Anatomical Therapeutic Chemical classification codes) and discharge summaries of hospitalisation records (based on free-text trade and generic names). See Online Resource 1 for detailed search terms and codes.

The start of the observation period for each individual was defined as their first recorded GP consultation, prescription or hospitalisation within the dataset. For the oral bisphosphonate cohort, as it is a first-line treatment, they were defined as “newly prescribed” if they received a first prescription for a bisphosphonate and had at least 12 months of observation before this without receiving any bone-health medication prescription (see Online Resource 1 for definition and codes). For the denosumab cohort, they were defined as “newly prescribed” if they received a first denosumab prescription and had at least 12 months of observation before this without a denosumab prescription.

### Estimate of persistence

Persistence was defined as the time from initiation to discontinuation of therapy [13]. Discontinuation was considered to have occurred if there was a gap in coverage of prescriptions of more than 90 days. This grace period ensured patients with short periods of discontinuation (e.g. due to dental procedures), or delay in obtaining a new prescription was not classified as having discontinued. The coverage of prescriptions for oral bisphosphonates was calculated based on specified duration and number of issues detailed in GP prescription records, while each prescription of denosumab covered a 6-month period (168 days). For the small proportion of prescriptions that were based on hospital discharge summaries, a 6-month prescription (168 days) was assumed. All patients were observed for as long as data allowed after initiation of medication. For calculation of 2-year persistence, patients were excluded if the initiation of medication occurred less than 2 years before the end of the data collection period. The number of patients who switched to an alternative bone-health medication within 90 days at the end of coverage period of the initial medication was calculated. These patients were subsequently excluded from the estimate of 2-year persistence. A sensitivity analysis was conducted to include those who switched in estimating 2-year persistence, adding persistence to their new medication to persistence to their initial medication.

#### Statistical analysis

Demographic and clinical variables were described for bisphosphonate and denosumab cohorts. Two-year persistence for bisphosphonates and denosumab was calculated with 95% confidence intervals.

### Factors associated with time to discontinuation

Time to discontinuation of medication was calculated in days for oral bisphosphonates and for denosumab for each patient who had at least 12 months of data after medication initiation. Patients who were found to switch to an alternative bone-health medication were excluded from time to discontinuation analysis.

#### Exposures

Exposures were defined during the time period prior to medication initiation. These included age at the point of medication initiation, a record of osteoporosis, fragility fracture or calcium/ vitamin prescription in GP or hospitalisation records (Online Resource 1), number of unique prescribed medications in the 12 months prior to initiation and health cover type. Number of medications was analysed categorically (0–5, 6–10, 11–15 and >15 medications). Health cover type was grouped into three categories relevant to the Irish healthcare system based on whether patients are required to pay at the point of care: “general medical services scheme” (GMS, covering GP care, hospital care and medications), doctor visit card (DVC, covering GP care only) and private [27]. For oral bisphosphonates, dosing frequency of medication (weekly or monthly) was also included as an exposure. For the denosumab cohort, whether the patient had been on previous bone-health medication was included in the analysis.

#### Statistical analysis

Separate Kaplan-Meier curves were generated to explore time to discontinuation of oral bisphosphonates (by dosing frequency) and denosumab. Factors associated with time to discontinuation were explored using univariable and multivariable Cox regression for oral bisphosphonates and denosumab. Unadjusted and adjusted hazard ratios (HRs) were calculated with 95% confidence intervals (CIs). Confidence intervals were adjusted for clustering of patients within GP practices. Stata 16 (StataCorp. 2019) was used for analyses and statistical significance was assumed at *p*<0.05.

## Results

### Participants

Figure 1 shows a flow diagram of selected patients. From 41,901 patients, *n*=1569 newly initiated on oral bisphosphonates and *n*=1615 on denosumab. Characteristics of the cohorts are presented in Table 1. The majority of prescriptions were identified from GP records rather than hospital discharge summaries. In the bisphosphonate cohort, 89% (*n*=1391) were prescribed a medication with a weekly regimen, while 11% (*n*=178) were prescribed monthly dosing frequencies. In the denosumab cohort, *n*=689 individuals (43% of those who initiated) had been prescribed a different bone-health medication previously, while *n*=926 (57%) were observed to initiate directly onto denosumab. In total, 56% and 51% of the bisphosphonate and denosumab cohorts were observed to discontinue the medication. Only 9% and 6% of those who discontinued bisphosphonates and denosumab, respectively, were switched to a different bone-health medication within 90 days at the end of the coverage period (Table 1).

**Table 1:** Cohort characteristics

	Bisphosphonates cohort (*n*=1569)	Denosumab cohort (*n*=1615)
Age (mean (SD))	76.6 (SD=8.1)	78.4 (SD=8.2)
Female sex (*n* (%))	1242 (79.3%)	1463 (90.6%)
Health cover:		
Private and others	270 (17.2%)	263 (16.3%)
GMS	1156 (73.7%)	1157 (71.6%)
DVC	142 (9.1%)	195 (12.1%)
Osteoporosis diagnosis	470 (30.0%)	767 (47.5%)
Fracture history	157 (10.0%)	185 (11.5%)
Calcium/vitamin D prescription	1294 (82.5%)	1415 (87.6%)
Source of prescription:		
Hospitalisation records	178 (11.3%)	75 (4.6%)
General practice prescriptions	1391 (88.7%)	1540 (95.4%)
Months between medication initiation and end of data collection period (mean (SD))	41.4 (SD=16.2)	36.9 (SD=15.6)
Discontinued medication (*n* (%))	882 (56.3%)	829 (51.3%)
Switched to alternative bone-health medication (% of discontinued)	81 (9.2%)	47 (5.7%)
Replacement medication:		
Oral bisphosphonates (*n*)	N/A	39
Zoledronate (*n*)	0	0
Denosumab (*n*)	78	N/A
Raloxifene (*n*)	0	2
Parathyroid hormone (*n*)	0	3
Strontium (*n*)	3	3

### Estimate of persistence

For oral bisphosphonates and denosumab, *n*=1212 and *n*=1146 patients, respectively, had at least 2 years between medication initiation and the end of data collection and did not switch to an alternative bone-health medication. Among these groups, 2-year persistence was 49.4% (95% CI 46.5% to 52.2%) for bisphosphonates and 53.8% (95% CI 50.9% to 56.8%) for denosumab. Sensitivity analysis including those who switched to an alternative medication resulted in estimates of 50.9% (95% CI 48.2% to 53.7%) for bisphosphonates and 53.6% (95% CI 50.7% to 56.4%) for denosumab.

### Factors associated with time to discontinuation of oral bisphosphonates

**Fig. 2:**
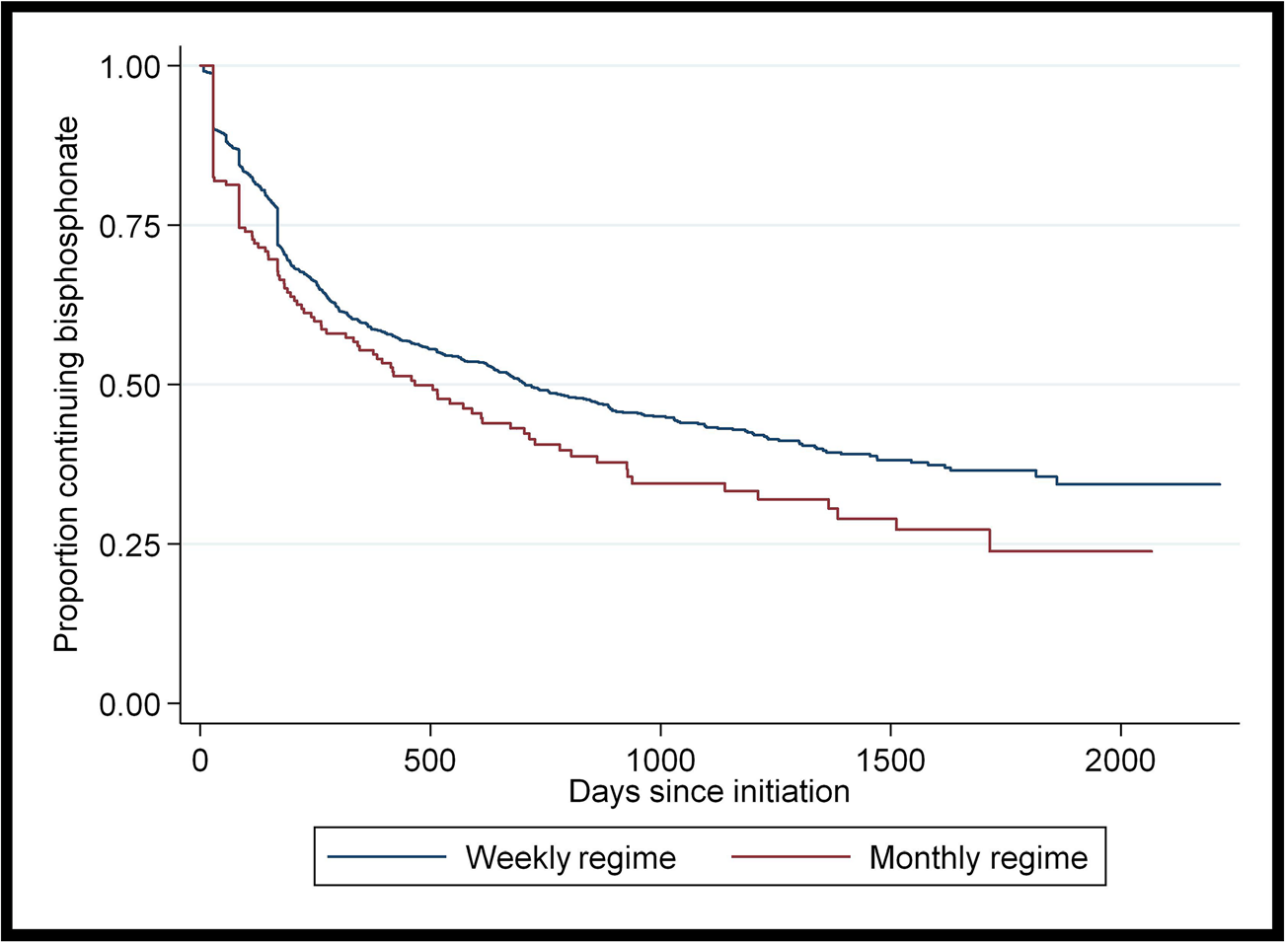
Kaplan-Meier graph of time to discontinuation of bisphosphonates by dosing frequency

A total of *n*=1487 patients were included in the time to discontinuation of oral bisphosphonates analysis. In the *n*=801 patients who discontinued bisphosphonates without switching onto another bone-health medication, mean time to discontinuation was 295 days (SD=332 days). Figure 2 shows a Kaplan-Meier graph of time to discontinuation of bisphosphonates by dosing frequency. Those on monthly regimens had a higher risk of discontinuation (log-rank test, *p*=0.02).

On multivariable analysis (Table 2), being 80 years or older (HR=1.26, 95% CI=1.04 to 1.52, *p*=0.02) was associated with a higher hazard of discontinuation of oral bisphosphonates. GMS health cover (HR=0.49, 95% CI=0.36 to 0.66, *p*<0.01), prescription of calcium or vitamin D (HR= 0.79 95% CI= 0.66 to 0.93, *p*<0.01) and being on 6–10 medications rather than 0–5 medications (HR= 0.82 95% CI= 0.69 to 0.98, *p*=0.03) were associated with a lower hazard of discontinuation of oral bisphosphonates. The relationship between time to discontinuation and oral bisphosphonate dosing frequency did not remain statistically significant on multivariable analysis.

**Table 2:** Factors associated with time to discontinuation of oral bisphosphonates (*n*=1487)

			Univariable			Multivariable		
	Maintained (*n*=686)	Discontinued (*n*=801)	HRR	95% CI	*p*-value	HRR	95% CI	*p*-value
Age group at initiation:								
<70 years	158 (23.0%)	188 (23.5%)	REF					
70–79 years	266 (38.8%)	317 (39.6%)	0.99	0.82 to 1.18	0.88	1.16	0.94 to 1.44	0.17
≥80 years	262 (38.2%)	296 (37.0%)	1.01	0.81 to 1.25	0.95	1.26	1.04 to 1.52	0.02*
Sex (% female)	527 (76.9 %)	640 (80.0%)	1.04	0.85 to 1.28	0.69	1.04	0.83 to 1.3	0.71
Health cover:								
Private and Other	98 (14.3%)	160 (20%)	REF					
GMS	562 (81.9%)	528 (66%)	0.49	0.38 to 0.64	<0.01*	0.49	0.36 to 0.66	<0.01*
DVC	26 (3.8%)	112 (14%)	1.18	0.86 to 1.63	0.30	1.11	0.78 to 1.57	0.57
Osteoporosis diagnosis	211 (30.8%)	229 (28.6%)	0.90	0.73 to 1.09	0.27	0.86	0.70 to 1.05	0.14
Fracture history	82 (12%)	67 (8.4%)	0.82	0.64 to 1.04	0.11	0.82	0.64 to 1.05	0.11
Calcium/vitamin D prescription	589 (85.9%)	634 (79.2%)	0.74	0.62 to 0.89	<0.01*	0.79	0.66 to 0.93	<0.01*
Number medications in previous 12 months:								
0 to 5	151 (22%)	252 (31.5%)	REF					
6 to 10	217 (31.6%)	250 (31.2%)	0.71	0.60 to 0.85	<0.01*	0.82	0.69 to 0.98	0.03*
11 to 15	177 (25.8%)	158 (19.7%)	0.64	0.49 to 0.83	<0.01*	0.77	0.59 to 1.01	0.06
> 15	141 (20.6%)	141 (17.6%)	0.70	0.57 to 0.85	<0.01*	0.83	0.66 to 1.05	0.12
Dosing frequency:								
Weekly	624 (91%)	697 (87%)	REF					
Monthly	62 (9%)	104 (13%)	1.28	1.03 to 1.59	0.02*	1.21	0.97 to 1.50	0.09

*GMS* general medical services scheme, *DVC* doctor visit card

*p<0.05

### Factors associated with time to discontinuation of denosumab

**Fig. 3:**
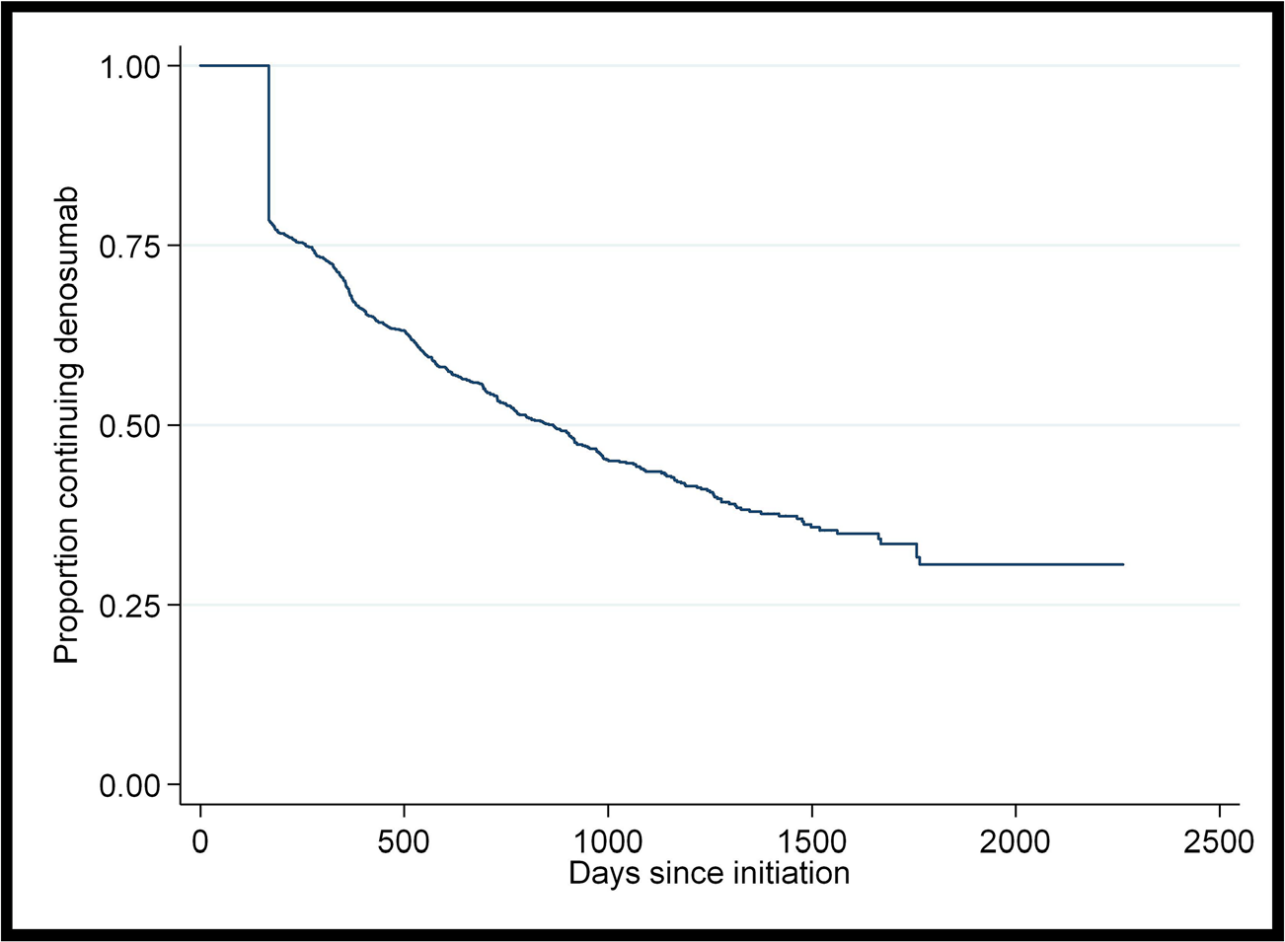
Kaplan-Meier graph of time to discontinuation of denosumab

A total of *n*=1568 patients were included in the time to discontinuation of denosumab analysis. In the *n*=782 patients who discontinued denosumab without switching onto another bone-health medication, mean time to discontinuation was 401 days (SD=321 days). Figure 3 shows a Kaplan-Meier graph of time to discontinuation of denosumab.

On multivariable analysis (Table 3), no factors were found to be associated with a higher hazard of discontinuation of denosumab. GMS health cover (HR=0.71 95% CI= 0.57 to 0.89, *p*<0.01) and having a diagnosis of osteoporosis (HR= 0.76 95% CI= 0.69 to 0.84, *p*<0.01) were associated with a lower hazard of discontinuation of denosumab.

**Table 3:** Factors associated with time to discontinuation of denosumab (*n*= 1568)

			Univariable			Multivariable		
	Maintained (*n*=786)	Discontinued (*n*=782)	HRR	95% CI	*p*-value	HRR	95% CI	*p*-value
Age group at initiation:								
<70 years	121 (15.4%)	161 (20.6%)						
70–79 years	291 (37.0%)	261 (33.4%)	0.82	0.71 to 0.96	0.01*	0.91	0.75 to 1.11	0.36
≥80 years	374 (47.6%)	360 (46.0%)	0.93	0.80 to 1.07	0.32	1.05	0.86 to 1.27	0.65
Sex (% female)	712 (90.6%)	707 (90.4%)	0.83	0.68 to 1.03	0.09	0.89	0.72 to 1.09	0.26
Health cover:								
Private and others	122 (15.5%)	133 (17%)	REF					
GMS	582 (74.1%)	538 (68.8%)	0.71	0.59 to 0.87	<0.01*	0.71	0.57 to 0.89	<0.01*
DVC	82 (10.4%)	111 (14.2%)	0.90	0.73 to 1.11	0.34	0.9	0.69 to 1.16	0.41
Osteoporosis diagnosis	402 (51.2%)	341 (43.6%)	0.75	0.68 to 0.82	<0.01*	0.76	0.69 to 0.84	<0.01*
Fracture history	106 (13.5%)	77 (9.9%)	0.80	0.61 to 1.06	0.13	0.83	0.62 to 1.11	0.21
Calcium/vitamin D prescription	696 (88.6%)	676 (86.5%)	0.88	0.75 to 1.04	0.13	0.96	0.81 to 1.14	0.66
Number medications in previous 12 months:								
0 to 5	138 (17.6%)	149 (19.1%)	REF					
6 to 10	221 (28.1%)	227 (29%)	0.97	0.82 to 1.15	0.71	1.04	0.87 to 1.25	0.68
11 to 15	218 (27.7%)	229 (29.3%)	0.97	0.82 to 1.14	0.69	1.02	0.86 to 1.19	0.85
>15	209 (26.6%)	177 (22.6%)	0.91	0.78 to 1.07	0.25	0.96	0.82 to 1.13	0.62
Previous bone-health medication use	335 (42.6%)	326 (41.7%)	0.90	0.81 to 1.01	0.07	0.95	0.85 to 1.08	0.45

*GMS* general medical services scheme, *DVC* doctor visit card

*p<0.05

## Discussion

### Summary of findings

This study includes a large and representative cohort of older adults in the primary care setting with a long period of follow-up between 2012 and 2017. To our knowledge, it is the first estimate of persistence in bone-health medication in a general older population in the Republic of Ireland since 2009 and the widespread introduction of denosumab [11, 28]. Findings of suboptimal 2-year persistence (49% for oral bisphosphonates and 54% for denosumab) in the current study are in line with previous research [13, 14]. Having state-funded health cover was the only factor found to be protective against discontinuation of both medications.

### Findings in the context of previous research

For over half of patients in this study who were started on denosumab, it was the first bone-health medication they were observed to take, despite it not being recommended as a first-line treatment in most cases [6–8, 21]. This recommendation is due in part to the cost of the medication but also due to the need to pre-screen for hypocalcaemia and comorbidities and due to complications that arise with cessation of the drug [7, 21, 29]. This pattern of prescribing reflects findings from a large primary care study in Australia where denosumab went from making up a small percentage of bone-health prescriptions in 2012 to being the most frequently prescribed in 2017 [23]. The denosumab cohort in this study included a higher proportion of female patients and more patients with a diagnosis of osteoporosis in comparison to the bisphosphonate cohort. This aligns with the strength of evidence for denosumab among women at highest risk of fracture [5, 7, 9]. The rate of contraindication to oral bisphosphonates among this group is not known; however, it is unlikely to explain the rate of denosumab prescribing as first-line therapy. Due the increased popularity of the medication among GPs in recent years, further investigation of the reasoning behind these treatment decisions is warranted.

A particularly concerning finding is that only 6% of those who discontinued on denosumab were switched to an alternative bone-health medication despite this being strongly recommended by current evidence [22]. For those not switched to another medication, only 55% continued taking denosumab without a gap in treatment for 2 years, which is similar to findings of a recent systematic review including 16 studies from the USA, Canada and sixteen European countries [14]. Where denosumab injections are received 9–12 months apart as opposed to 6 monthly, bone turnover markers increase significantly, while increases in BMD drop by over half [18, 19]. A post hoc analysis of a randomised controlled trial of 1001 participants also found the rate of vertebral fractures increases five-fold on discontinuation of denosumab, quickly approaching the fracture rate observed on placebo [20]. While switching to oral bisphosphonates can protect against these changes in most patients, recently published results of a randomised controlled trial of 61 patients on longer-term therapy found that a single dose of zoledronate infusion was not sufficient to maintain benefits [21, 22]. GPs in Australia have expressed an awareness of the quick reversal of BMD gains after stopping denosumab but also uncertainty about how and when to stop denosumab or the risks of doing so [23]. It is likely that GPs in Ireland have similar concerns and education and support in this area appears to be urgently required.

Poor persistence on oral bisphosphonates is also a concern. A 2011 meta-analysis of five studies and over 100,000 patients indicated that fracture risk increased by up to 40% with non-persistence of bisphosphonates [12]. In the oral bisphosphonate cohort in the current study, 2-year persistence was estimated at 49% between 2012 and 2017, showing no improvement on older work [11, 28]. Two previous Irish studies of bisphosphonate persistence between 2005 and 2009 showed a 1-year rate of less than 50% in patients hospitalised with a fragility fracture [28] and a 2-year rate of 50% in the general older population [11]. In five studies from the USA, Canada, Hungary and Sweden that were included in a recent systematic review and that measured 2-year persistence using similar treatment gaps as the current analysis, estimates ranged from 19 to 46% [13, 30–34]. While there is some clinical uncertainty about whether particular patients should be given a break or “bisphosphonate holiday” after 3–5 years to avoid increasing the risk of adverse events, this should not influence persistence after only 2 years on medication [15–17, 23]. Furthermore, this cohort would be considered at relatively higher risk of fragility fracture as they have an average age of 77, a fracture history prevalence of 10% and a diagnosis of osteoporosis in 30% of the group. Guidelines suggest that among patients at high fracture risk, alendronic acid may be safely continued for up to 10 years and risedronate for up to 7 years [6]. It should be noted that our estimate of persistence could be optimistic as we used a conservative acceptable treatment gap of 90 days and excluded switchers onto alternative medications [13, 35]. In addition, in contrast to previous research, our study did not find more frequent dosing regimens of oral bisphosphonates to be associated with discontinuation [28, 31–33, 36]. In fact, on univariable analysis, monthly regimens had a higher hazard ratio than weekly formulations. This may reflect monthly formulations being targeted towards patients likely to have challenges persisting to the prescribed regimen.

Having state-funded health cover (GMS) was the only factor found to be protective against discontinuation of both oral bisphosphonates and denosumab in this study. This relationship remained strong even after adjusting for age. This is important, as the GMS scheme in Ireland is means-tested but a higher income threshold applies to those aged 70 and over [27]. For this reason, 50–55% of patients in this study aged under 70 years were covered by DVC/GMS in comparison to 90% of patients 70 years and older. Such patients who have free access to GPs, practice nurses and medications may be more likely to return for repeat prescriptions, support and administration of medication (in the case of denosumab). Older age group (80 years and older) showed some association with discontinuation of oral bisphosphonates on multivariable analysis, independent of health cover, but this was not observed in the denosumab cohort. In previous literature, age has shown an inconsistent relationship with persistence of these medications with both the oldest (>75 years) and youngest (<65 years) most likely to discontinue [13, 35, 37, 38]. This may be related to an increased likelihood of adverse effects at older ages or patients or physicians not prioritising treatment of fracture risk in younger patients [35]. As this study included only patients who were aged over 65 by 2017, this could have resulted in higher persistence overall.

In our study, prescription of calcium or vitamin D was associated with a lower hazard of discontinuation of oral bisphosphonates, and having a recorded diagnosis of osteoporosis was associated with a lower hazard of discontinuation of denosumab. This could potentially be explained by ongoing osteoporosis management being reflective of the patient and physician prioritising the need for therapy, which is suggested to be an important determinant of adherence to these medications [35]. Prior BMD testing, using other drugs for osteoporosis and calcium or vitamin D supplementation has been associated with better persistence in previous research [13, 36, 39]. In contrast to several other studies, however, a history of fragility fracture was not found to be associated with improved persistence in our analysis [13, 39]. This is surprising, as one would expect a fracture experience to highlight the need for treatment and improve the management pathway. Studies in Australia and Canada have found that for secondary fracture prevention, while specialist-led programmes can facilitate better initiation of therapy, primary care physician follow-up is as effective at improving persistence [40, 41]. This suggests that GPs could be supported to provide long-term management of osteoporosis in patients with fragility fracture but that once-off reviews with geriatricians could be beneficial. This requires further investigation in the Irish setting.

### Strengths and limitations

A strength of this study is that we ascertained prescribing from multiple sources (i.e. GP prescription records and hospitalisation discharge summaries). We included a washout period to look at those newly initiated on medication as patients have been found to be more persistent if evaluated from their first exposure to osteoporosis therapy [42]. Using routinely collected data, we were unable to assess reasons for discontinuation of medication that may have been clinically appropriate and do not know if it resulted from a risk-benefit discussion with patients. Therefore some cases of non-persistence may have been discontinuations for a clinically appropriate reason. We were also unable to determine if patients received prescriptions/ treatment (including denosumab or bisphosphonate infusion) solely from outpatient appointments with hospital-based specialists or during hospital admissions. Regardless, the very high rate of non-persistence to denosumab without observed replacement by bisphosphonates or other therapies within the primary care setting is a major concern, due to risk of rebound vertebral fractures [20]. As data related to prescribing, it is not possible to determine whether prescriptions were dispensed or if patients took their medication as prescribed. This may have resulted in an optimistic persistence rate. Finally, it is unknown, whether those “initiated” could have been finishing a “bisphosphonate holiday” or those discontinuing could have re-initiated later on. Discontinuing denosumab however has significant risks in the short-term, and so looking for delayed re-initiations was not an objective of our analysis.

### Clinical implications

Non-persistence/adherence to osteoporosis medications is wasteful and can pose significant patient risks, especially in the case of denosumab treatment. Further research is required in the Irish primary care setting, given the mixed public-private health system, to explore the reasons for prescribing choices and patterns and to evaluate interventions targeted at both patients and physicians. A recent systematic review found that multi-component education programmes that included patients in the decision-making process around osteoporosis treatment and specific regimens improved medication persistence [43]. A 2012 Irish analysis suggested that investing €120 annually per patient into interventions would remain cost-effective if they improved adherence and persistence to osteoporosis medication by just 10% [11]. This warrants further testing.

## Conclusion

This study has identified a number of areas where fracture preventive prescribing among older adults in primary care could be improved. This includes the common use of denosumab as a first-line treatment, suboptimal rates of persistence with bisphosphonates and denosumab at 2 years and low rates of switching to other preventative treatments among those stopping denosumab. Free access to primary care services and medications may facilitate persistence; however, other interventions targeting patients and prescribing in primary care to optimise prescribing warrant evaluation.

## Supplementary information

ESM 1 (DOCX 16.9 kb)

## Data Availability

No additional data available.

## References

[1] Downey C, Kelly M, Quinlan JF (2019). Changing trends in the mortality rate at 1-year post hip fracture - a systematic review. World J Orthop.

[2] Kanis JA, on behalf of the World Health Organization Scientific Group (2007) Assessment of osteoporosis at the primary health-care level. In: World Health Organization Collaborating Centre for Metabolic Bone Diseases (ed) University of Sheffield, UK

[3] Wright NC, Looker AC, Saag KG, Curtis JR, Delzell ES, Randall S, Dawson-Hughes B (2014). The recent prevalence of osteoporosis and low bone mass in the United States based on bone mineral density at the femoral neck or lumbar spine. J Bone Miner Res.

[4] Amin S, Achenbach SJ, Atkinson EJ, Khosla S, Melton LJ (2014). Trends in fracture incidence: a population-based study over 20 years. J Bone Miner Res.

[5] Compston J, Cooper A, Cooper C (2017). UK clinical guideline for the prevention and treatment of osteoporosis. Arch Osteoporos.

[6] Scottish Intercollegiate Guideline Network (2015) Management of osteoporosis and the prevention of fragility fractures (SIGN Guideline No. 142). Edinburgh

[7] Davis S, Simpson E, Hamilton J, James MM, Rawdin A, Wong R, Goka E, Gittoes N, Selby P (2020). Denosumab, raloxifene, romosozumab and teriparatide to prevent osteoporotic fragility fractures: a systematic review and economic evaluation. Health Technol Assess.

[8] National Institute for Health and Care Excellence (2014) Osteoarthritis: care and management. NICE guideline (CG177) 31869054

[9] Nayak S, Greenspan SL (2017). Osteoporosis treatment efficacy for men: a systematic review and meta-analysis. J Am Geriatr Soc.

[10] Langdahl BL, Teglbjærg CS, Ho PR, Chapurlat R, Czerwinski E, Kendler DL, Reginster JY, Kivitz A, Lewiecki EM, Miller PD, Bolognese MA, McClung MR, Bone HG, Ljunggren Ö, Abrahamsen B, Gruntmanis U, Yang YC, Wagman RB, Mirza F, Siddhanti S, Orwoll E (2015). A 24-month study evaluating the efficacy and safety of denosumab for the treatment of men with low bone mineral density: results from the ADAMO trial. J Clin Endocrinol Metab.

[11] Hiligsmann M, McGowan B, Bennett K, Barry M, Reginster JY (2012). The clinical and economic burden of poor adherence and persistence with osteoporosis medications in Ireland. Value Health.

[12] Ross S, Samuels E, Gairy K, Iqbal S, Badamgarav E, Siris E (2011). A meta-analysis of osteoporotic fracture risk with medication nonadherence. Value Health.

[13] Fatoye F, Smith P, Gebrye T, Yeowell G (2019). Real-world persistence and adherence with oral bisphosphonates for osteoporosis: a systematic review. BMJ Open.

[14] Koller G, Goetz V, Vandermeer B, Homik J, McAlister FA, Kendler D, Ye C (2020). Persistence and adherence to parenteral osteoporosis therapies: a systematic review. Osteoporos Int.

[15] Eastell R, Rosen CJ, Black DM, Cheung AM, Murad MH, Shoback D (2019). Pharmacological management of osteoporosis in postmenopausal women: an endocrine society* clinical practice guideline. J Clin Endocrinol Metab.

[16] Camacho PM, Petak SM, Binkley N, Clarke BL, Harris ST, Hurley DL, Kleerekoper M, Lewiecki EM, Miller PD, Narula HS, Pessah-Pollack R, Tangpricha V, Wimalawansa SJ, Watts NB (2016). American association of clinical endocrinologists and American college of endocrinology clinical practice guidelines for the diagnosis and treatment of postmenopausal osteoporosis - 2016. Endocr Pract.

[17] Anagnostis P, Paschou SA, Mintziori G, Ceausu I, Depypere H, Lambrinoudaki I, Mueck A, Pérez-López FR, Rees M, Senturk LM, Simoncini T, Stevenson JC, Stute P, Trémollieres FA, Goulis DG (2017). Drug holidays from bisphosphonates and denosumab in postmenopausal osteoporosis: EMAS position statement. Maturitas..

[18] Lyu H, Zhao SS, Yoshida K, Tedeschi SK, Xu C, Nigwekar SU, Leder BZ, Solomon DH (2020). Delayed denosumab injections and bone mineral density response: an electronic health record-based study. J Clin Endocrinol Metab.

[19] Bone HG, Bolognese MA, Yuen CK, Kendler DL, Miller PD, Yang YC, Grazette L, San Martin J, Gallagher JC (2011). Effects of denosumab treatment and discontinuation on bone mineral density and bone turnover markers in postmenopausal women with low bone mass. J Clin Endocrinol Metab.

[20] Cummings SR, Ferrari S, Eastell R, Gilchrist N, Jensen JEB, McClung M, Roux C, Törring O, Valter I, Wang AT, Brown JP (2018). Vertebral fractures after discontinuation of denosumab: a post hoc analysis of the randomized placebo-controlled FREEDOM trial and its extension. J Bone Miner Res.

[21] Sølling AS, Harsløf T, Langdahl B (2020). Treatment with zoledronate subsequent to denosumab in osteoporosis: a randomized trial. J Bone Miner Res.

[22] Tsourdi E, Langdahl B, Cohen-Solal M, Aubry-Rozier B, Eriksen EF, Guañabens N, Obermayer-Pietsch B, Ralston SH, Eastell R, Zillikens MC (2017). Discontinuation of denosumab therapy for osteoporosis: a systematic review and position statement by ECTS. Bone..

[23] Naik-Panvelkar P, Norman S, Elgebaly Z, Elliott J, Pollack A, Thistlethwaite J, Weston C, Seibel MJ (2020). Osteoporosis management in Australian general practice: an analysis of current osteoporosis treatment patterns and gaps in practice. BMC Fam Pract.

[24] Benchimol EI, Smeeth L, Guttmann A, Harron K, Moher D, Petersen I, Sorensen HT, von Elm E, Langan SM (2015). The REporting of studies Conducted using Observational Routinely-collected health Data (RECORD) statement. PLoS Med.

[25] Redmond P, McDowell R, Grimes TC, Boland F, McDonnell R, Hughes C, Fahey T (2019). Unintended discontinuation of medication following hospitalisation: a retrospective cohort study. BMJ Open.

[26] Walsh ME, Nerdrum M, Fahey T, Moriarty F (2021) Factors associated with initiation of bone-health medication among older adults in primary care in Ireland. Age and Ageing 1–8. 10.1093/ageing/afab033. 10.1093/ageing/afab033 PMC8437061 33693466

[27] Health Service Executive (2019) Medical Card and GP Visit Card National Assessment Guidelines. Version 3

[28] McGowan B, Bennett K, Casey MC, Doherty J, Silke C, Whelan B (2013). Comparison of prescribing and adherence patterns of anti-osteoporotic medications post-admission for fragility type fracture in an urban teaching hospital and a rural teaching hospital in Ireland between 2005 and 2008. Ir J Med Sci.

[29] Albert SG, Reddy S (2017). Clinical evaluation of cost efficacy of drugs for treatment of osteoporosis: a meta-analysis. Endocr Pract.

[30] LeBlanc ES, Rosales AG, Balasubramanian A, O'Malley CD, Egbuna O, Friess D, Perrin NA (2015). Risk factors for fracture among current, persistent users of bisphosphonates. Osteoporos Int.

[31] Landfeldt E, Ström O, Robbins S, Borgström F (2012). Adherence to treatment of primary osteoporosis and its association to fractures--the Swedish Adherence Register Analysis (SARA). Osteoporos Int.

[32] Lakatos P, Takács I, Marton I, Tóth E, Zoltan C, Lang Z, Psachoulia E, Intorcia M (2016). A retrospective longitudinal database study of persistence and compliance with treatment of osteoporosis in Hungary. Calcif Tissue Int.

[33] Curtis JR, Westfall AO, Allison JJ, Freeman A, Saag KG (2006). Channeling and adherence with alendronate and risedronate among chronic glucocorticoid users. Osteoporos Int.

[34] Burden AM, Paterson JM, Solomon DH, Mamdani M, Juurlink DN, Cadarette SM (2012). Bisphosphonate prescribing, persistence and cumulative exposure in Ontario, Canada. Osteoporos Int.

[35] Hiligsmann M, Cornelissen D, Vrijens B, Abrahamsen B, al-Daghri N, Biver E, Brandi ML, Bruyère O, Burlet N, Cooper C, Cortet B, Dennison E, Diez-Perez A, Gasparik A, Grosso A, Hadji P, Halbout P, Kanis JA, Kaufman JM, Laslop A, Maggi S, Rizzoli R, Thomas T, Tuzun S, Vlaskovska M, Reginster JY (2019). Determinants, consequences and potential solutions to poor adherence to anti-osteoporosis treatment: results of an expert group meeting organized by the European Society for Clinical and Economic Aspects of Osteoporosis, Osteoarthritis and Musculoskeletal Diseases (ESCEO) and the International Osteoporosis Foundation (IOF). Osteoporos Int.

[36] Cotté FE, Fardellone P, Mercier F, Gaudin AF, Roux C (2010). Adherence to monthly and weekly oral bisphosphonates in women with osteoporosis. Osteoporos Int.

[37] Fahrleitner-Pammer A, Papaioannou N, Gielen E, Feudjo Tepie M, Toffis C, Frieling I, Geusens P, Makras P, Boschitsch E, Callens J, Anastasilakis AD, Niedhart C, Resch H, Kalouche-Khalil L, Hadji P (2017). Factors associated with high 24-month persistence with denosumab: results of a real-world, non-interventional study of women with postmenopausal osteoporosis in Germany, Austria, Greece, and Belgium. Arch Osteoporos.

[38] van der Zwaard BC, van Hout W, Hugtenburg JG, van der Horst HE, Elders PJM (2017). Adherence and persistence of patients using oral bone sparing drugs in primary care. Fam Pract.

[39] Martín-Merino E, Huerta-Álvarez C, Prieto-Alhambra D, Montero-Corominas D (2017). Cessation rate of anti-osteoporosis treatments and risk factors in Spanish primary care settings: a population-based cohort analysis. Arch Osteoporos.

[40] Ganda K, Schaffer A, Pearson S, Seibel MJ (2014). Compliance and persistence to oral bisphosphonate therapy following initiation within a secondary fracture prevention program: a randomised controlled trial of specialist vs. non-specialist management. Osteoporos Int.

[41] McAlister FA, Ye C, Beaupre LA, Rowe BH, Johnson JA, Bellerose D, Hassan I, Majumdar SR (2019). Adherence to osteoporosis therapy after an upper extremity fracture: a pre-specified substudy of the C-STOP randomized controlled trial. Osteoporos Int.

[42] Morley J, Moayyeri A, Ali L, Taylor A, Feudjo-Tepie M, Hamilton L, Bayly J (2020). Persistence and compliance with osteoporosis therapies among postmenopausal women in the UK Clinical Practice Research Datalink. Osteoporos Int.

[43] Cornelissen D, de Kunder S, Si L, Reginster JY, Evers S, Boonen A, Hiligsmann M (2020). Interventions to improve adherence to anti-osteoporosis medications: an updated systematic review. Osteoporos Int.

